# Biofilm Formation and Heat Stress Induce Pyomelanin Production in Deep-Sea *Pseudoalteromonas* sp. SM9913

**DOI:** 10.3389/fmicb.2017.01822

**Published:** 2017-09-21

**Authors:** Zhenshun Zeng, Xingsheng Cai, Pengxia Wang, Yunxue Guo, Xiaoxiao Liu, Baiyuan Li, Xiaoxue Wang

**Affiliations:** ^1^Key Laboratory of Tropical Marine Bio-resources and Ecology, The South China Sea Institute of Oceanology, Chinese Academy of Sciences Guangzhou, China; ^2^Guangdong Provincial Key Laboratory of Microbial Culture Collection and Application, Guangdong Institute of Microbiology Guangzhou, China

**Keywords:** pyomelanin, temperature, *Pseudoalteromonas*, biofilm, pellicle formation

## Abstract

*Pseudoalteromonas* is an important bacterial genus present in various marine habitats. Many strains of this genus are found to be surface colonizers on marine eukaryotes and produce a wide range of pigments. However, the exact physiological role and mechanism of pigmentation were less studied. *Pseudoalteromonas* sp. SM9913 (SM9913), an non-pigmented strain isolated from the deep-sea sediment, formed attached biofilm at the solid–liquid interface and pellicles at the liquid–air interface at a wide range of temperatures. Lower temperatures and lower nutrient levels promoted the formation of attached biofilm, while higher nutrient levels promoted pellicle formation of SM9913. Notably, after prolonged incubation at higher temperatures growing planktonically or at the later stage of the biofilm formation, we found that SM9913 released a brownish pigment. By comparing the protein profile at different temperatures followed by qRT-PCR, we found that the production of pigment at higher temperatures was due to the induction of *melA* gene which is responsible for the synthesis of homogentisic acid (HGA). The auto-oxidation of HGA can lead to the formation of pyomelanin, which has been shown in other bacteria. Fourier Transform Infrared Spectrometer analysis confirmed that the pigment produced in SM9913 was pyomelanin-like compound. Furthermore, we demonstrated that, during heat stress and during biofilm formation, the induction level of *melA* gene was significantly higher than that of the *hmgA* gene which is responsible for the degradation of HGA in the L-tyrosine catabolism pathway. Collectively, our results suggest that the production of pyomelanin of SM9913 at elevated temperatures or during biofilm formation might be one of the adaptive responses of marine bacteria to environmental cues.

## Introduction

Biotic and abiotic surfaces in various marine environments are rapidly colonized by microorganisms, and surface colonization by marine microbes often involves biofilm formation, which aids survival in extreme marine environments (Dang and Lovell, 2016). During biofilm formation, marine bacteria often produced biologically active compounds to adapt the extreme environmental conditions, such as high pressure, hydrothermal vent, and depletion of micronutrients (de Carvalho and Fernandes, 2010). *Pseudoalteromonas* is a genus of *Gammaproteobacteria* that is widespread in the world’s ocean and have been shown to produce bioactive compounds that inhibit settling of several fouling invertebrates and algae during biofilm formation (Holmstrom and Kjelleberg, 1999; Egan et al., 2002; Ballestriero et al., 2010). Our recent work showed that biofilms formed by genetic variants of *Pseudoalteromonas lipolytica* with different capsular polysaccharide production or cellulose production varied with their ability to induce the larval settlement and metamorphosis of the mussel *Mytilus coruscus* (Zeng et al., 2015).

Production of the bioactive pigment is prevalent in marine bacteria and is important to cellular physiology and survival. Bioactive pigments with broad-ranging pharmacological activities has been received extensively studied, and it has been suggested that pigmented *Pseudoalteromonas* spp. produce more bioactive molecules (Bowman, 2007). *Pseudoalteromonas* spp. can be divided into pigmented and non-pigmented clades based on their ability to produce pigments. Of the twenty-three pigmented *Pseudoalteromonas* strains identified to date, three of them have been shown to produce melanin-like pigments, including *Pseudoalteromonas nigrifaciens* (Baumann et al., 1984), *Pseudoalteromonas aliena* (Ivanova et al., 2004), and *Pseudoalteromonas distinct* (Ivanova et al., 2004). A number of marine bacteria, such as *Vibrio cholerae* (Coyne and Alharthi, 1992), *Shewanella colwelliana* (Fuqua and Weiner, 1993), *Cellulophaga tyrosinoxydans* (Kahng et al., 2009), and *Marinomonas mediterranea* (Solano et al., 1997) also have been shown to produce dark-brown melanins. Melanins constitute a general class of complex polyphenolic heteropolymers that include eumelanin, pheomelanin, pyomelanin, and a number of pathways are known to covert tyrosine into melanin (Ito and Wakamatsu, 2003; Rzepka et al., 2016). Pyomelanin was produced by the accumulation of homogentisic acid (HGA), which is synthesized via 4-hydroxyphenylpyruvate dioxygenase (4-HDDP) in the process of L-tyrosine degradation; HGA is excreted to extracellular and auto-oxidized, followed by polymerized to form pyomelanin (Schmaler-Ripcke et al., 2009). The dark brown pigment synthesized by certain strains of *Vibrio cholerae* and *Shewanella colwelliana* is a type of pyomelanin derived from HGA (Coyne and Alharthi, 1992; Coon et al., 1994). Although melanin-like pigments are produced in different marine bacteria, little is known about the melanin precursors or biosynthetic enzymes as well as the regulation of melanin production in *Pseudoalteromonas* spp.

In this study, we studied a deep-sea derived non-pigmented *Pseudoalteromonas* sp. SM9913. SM9913 is a psychrotolerant bacterium and can grow at a wide range of temperatures from 4 to 37°C (Qin et al., 2007). Comparative genomic analysis of SM9913 with the surface sea-water psychrophilic bacterium of *Pseudoalteromonas haloplanktis* TAC125 has revealed some special genetic features that may have allowed it to adapt to the sediment-attached lifestyle (Qin et al., 2011). SM9913 has both the polar and lateral flagella systems, and produces a large quantity of exopolysaccharides and proteases, which favor degrading the sedimentary particulate organic nitrogen and adopting a particle-associated biofilm lifestyle in the sediment (Chen et al., 2003; Mi et al., 2015; Liu et al., 2016). In this study, we first assessed the biofilm formation of SM9913 under various conditions. SM9913 formed attached biofilm and pellicles at 4–37°C. Interestingly, we found that SM9913, considered to be a non-pigmented *Pseudoalteromonas* strain, produces a dark brown pigment when cultured at higher temperatures or when forming biofilm. We demonstrated that higher temperatures and biofilm formation induced pyomelanin production in SM9913 via accumulation of the *melA* gene product, 4-HPPD. Transcription of *melA* was induced more than three other genes (*hmgA*, *maiA*, and *fahA*) during the catabolism of L-tyrosine at higher temperatures and during biofilm formation, but not upon the addition of L-tyrosine to the culture medium at 15°C. Knockout *melA* gene in SM9913 completely abolished pyomelanin production when cultured at higher temperatures, while ectopic expression of *melA* in *melA* knockout strain restored pyomelanin production at higher temperatures. Finally, knockout of *melA* reduced resistance to heat stress in SM9913. These findings demonstrate that pyomelanin production may be one of the adaptive strategies of marine bacteria.

## Materials and Methods

### Bacterial Strains, Plasmids, and Growth Conditions

The bacterial strains and plasmids used in this study are listed in **Table 1**. *Pseudoalteromonas* sp. SM9913 was a generous gift from Professor Yuzhong Zhang at Shandong University, China, and was originally isolated from deep-sea sediment at a water depth of 1855 m near the Okinawa Trough (Chen et al., 2002). SM9913 was routinely cultured in a nutrient-enriched medium SWLB (seawater Luria-Bertani: 10 g peptone, 5 g yeast extract, 1 L artificial seawater) or a nutrient-less marine broth 2216E at 15°C (Becton, Dickinson and Company, Franklin Lakes, NJ, United States) (Qin et al., 2011). For *Escherichia coli*, experiments were conducted in LB medium at 37°C. When needed, antibiotics were added to the medium at the following concentrations: erythromycin at 25 μg/mL and chloramphenicol at 30 μg/mL. DAP (2, 6-diamino-pimelic acid) was added to the medium at a concentration of 0.3 mM to culture *E. coli* WM3064 strain.

**Table 1:** The bacterial strains and plasmids used in this study.

Strains and plasmids	Genotype/relevant characteristics	Source
***Pseudoalteromonas* strain**	
SM9913	Wild-type	Chen et al., 2002
Δ*melA*	SM9913 with the chromosomal *melA* gene deleted	This study
***E. coli* strains**	
K-12 BW25113	*lacI*^q^*rrnB*_T14_*DlacZ*_WJ16_*hsdR*514D*araBAD*_AH33_D*rhaBAD*_LD78_	Baba et al., 2006
WM3064	Donor strain for conjugation, Diaminopimelate (DAP) auxotrophic	Dehio and Meyer, 1997
**Plasmids**		
pCA24N	Cm^R^; *lacI*^q^	Kitagawa et al., 2005
pCA24N-*melA*	Cm^R^; *lacI*^q^, pCA24NP*_T5-lac_*::*melA*^+^, vector for expressing *melA* of SM9913 in *E. coli* host	This study
pBBR1MCS-Cm	pBBR1MCS2 with a chloramphenicol resistance gene inserted	Wang et al., 2015
pBBR1MCS-Cm-*melA*	PCR fragment carrying *melA* of SM9913 and the promoter region cloned into pBBR1MCS-Cm	This study
pK18*mobsacB*-Ery	Km^R^, Ery^R^, suicide vector for gene knockout	Wang et al., 2015


*Chloramphenicol (30 μg/mL) was used to maintain pCA24N-based and pBBR1MCS-Cm-based plasmids, while erythromycin (25 μg/mL) was used to maintain pK18mobsacB-Ery-based plasmids*.

### Attached Biofilm and Pellicle Formation Assay

Attached biofilm formation was assayed in 96-well polystyrene plates using crystal violet staining as described previously (Pratt and Kolter, 1998). Briefly, cells were grown in SWLB or 2216E medium without shaking at different temperatures. Attached biofilm was measured at the indicated time points. To remove the growth effects, biofilm formation was normalized by dividing the total biofilm by the maximal bacterial growth as measured by turbidity OD_620_ for each strain. Three independent cultures were used for each strain. To form the pellicle (air–liquid biofilm), cells were grown in SWLB or 2216E medium in glass beakers without shaking for the specified number of days at different temperatures. Pellicles were assayed by visual inspection of the air–liquid interface of the standing culture. Morphology was observed and photographed at the indicated time points.

### Pigment Production and Quantification

Wild-type SM9913 strain was grown in SWLB or 2216E medium with or without the L-tyrosine or D-tyrosine at a concentration of 1 mg/mL for different days. *E. coli* K12 carries the pCA24N-*melA* or empty vector were grown in LB medium supplemented with 30 μg/mL chloramphenicol and 0.5 mM IPTG (isopropyl-beta-D-thiogalactopyranoside) for 2 days. The production of pigment was observed and photographed at the indicated time points. To measure the pigment production, the cultures were centrifuged at the 17000 *g* for 15 min, and the supernatants were collected and quantified by measuring the absorbance at 400 nm (Turick et al., 2002).

### SDS-PAGE and Mass Spectrometry

Wild-type SM9913 strain was grown in SWLB medium at 15 or 37°C for 24 h, respectively. The cultures of 5 mL were centrifuged at 13,000 rpm for 2 min and the supernatants were removed. Cell pellets were re-suspended in 1 mL lysis buffer [50 mM Tris (pH 8.0), 100 mM NaCl, and protease inhibitor cocktail (Sigma–Aldrich, St. Louis, MO, United States)]. Then samples were sonicated twice at level 2 for 5 min using a Sonic Dismembrator (Ningbo Scientz Biotechnology, Co., Ltd., Ningbo, China). The supernatant was collected after centrifugation at 13,000 rpm for 5 min and the protein concentration was measured using a Bi Yuntian BCA assay kit (Beyotime Biotechnology, Co., Ltd., Haimen, China). Sodium dodecyl sulfate polyacrylamide gel electrophoresis (SDS-PAGE) was performed by loading 25 μg of each sample. Gels were stained with Coomassie Brilliant Blue R-250 and 10 protein bands were cut and subjected to in-gel tryptic digestion. In-gel digestion and mass spectrometry (MS) analysis of the gel slices were performed at the Research Center for Life Sciences University of Science and Technology (Hefei, Anhui, China) as previously described (Shevchenko et al., 2006).

### Fourier Transform Infrared Spectrometer Analysis

The pigment of SM9913 induced at 37°C was extracted according to a previously published method with minor modifications (Schmaler-Ripcke et al., 2009). Briefly, SM9913 wild-type cells were grown in SWLB medium at 37°C for 48 h. A 200 mL culture was collected and centrifuged at 13,000 rpm for 5 min. The supernatants were then filtrated through 0.45 μM filter membrane. The filtrate was acidified with 6 M HCl to a pH of 2.0, and it was allowed to precipitate overnight at room temperature. After centrifugation (16, 500 × *g*, 30 min), the pellet was re-suspended in 2.5 mL deionized water at pH of 12 and dialyzed in 3.5-kDa dialysis tubing for 24 h against deionized water. The dialyzed pigment was then dried using the vacuum centrifugal concentrator (Tokyo Rikakikai, Co., Ltd., Tokyo, Japan). Synthetic pyomelanin (HGA-melanin) was used as a control compound in Fourier Transform Infrared Spectrometer (FTIR) analysis. Synthetic pyomelanin was produced by auto-oxidation of a 10 mM HGA (Tokyo Chemical Industry, Co., Ltd., Tokyo, Japan) at pH 10 with constant stirring on a magnetic stirrer for 3 days (Thermo Fisher Scientific, Waltham, MA, United States). A 200 mL sample was collected and precipitated by adjusting the pH to 2 with 6 M HCl. Further treated is performed as described above. The pigment prepared from *in vitro* cultures of SM9913 and synthetic pyomelanin were analyzed by using FTIR spectrophotometer (IR Affinity-1, Kyoto, Japan).

### Construction of Strains and Vectors

In-frame deletion of a single gene in SM9913 was performed using the fusion PCR method we developed recently (Wang et al., 2015). Briefly, two primer pairs (melA-up-S/melA-up-A and melA-down-S/melA-down-A) were used to amplify the upstream and downstream of the target region from wild-type SM9913 genomic DNA. The resulting 842 and 836 bp fragments were digested with SphI/EcoRI and EcoRI/SalI, respectively, and then cloned into the SphI/SalI sites of the suicide plasmid pK18*mobsacB*-Ery. The constructed vector was then transformed into the *E. coli* WM3064 strain and verified by DNA sequencing using primer pair pK18-F/pK18-R. The recombinant suicide plasmid was mobilized from *E. coli* WM3064 into SM9913 by intergeneric conjugation at 15°C. Cells integrated the recombinant plasmid via a single crossover event was selected for by erythromycin resistance and confirmed by PCR using primer pair pK18-F/pK18-R. The deletion mutant was screened by plating the single-crossover strain on SWLB medium containing 15% sucrose. Further confirmation of the deletion mutant was carried out by PCR followed by DNA sequencing using primer pair melA-F/melA-R.

The broad-host-range plasmid pBBR1MCS-Cm was used for gene complementation (Wang et al., 2015). Gene fragment of *melA* was PCR amplified using SM9913 genomic DNA as template, and then inserted into the EcoRI/XhoI sites of pBBR1MCS-Cm. After verification by DNA sequencing using primer pair pBBR1MCS-F/pBBR1MCS-R, the expression plasmid was introduced into the Δ*melA* strain by conjugation. The plasmid pCA24N was used to express the *melA* product in *E. coli* K12. The *melA* gene was amplified from SM9913 genomic DNA using the primer pair pCA24N-*melA*-F/pCA24N-*melA*-R. The PCR product was phosphorylated, purified, and ligated into vector pCA24N as previously described (Guo et al., 2014). The recombinant plasmid was verified by DNA sequencing using primer pair pCA24N-F/pCA24N-R and introduced into *E. coli* K12. All the primers used in this study are listed in Supplementary Table S1.

### Quantitative Real-time Reverse-Transcription PCR (qRT-PCR)

Cells were grown at different temperatures and collected at the exponential stage (turbidity ∼1.0 at OD_600_) or during biofilm formation. To test the effect of the tyrosine on the genes transcription, cells were grown in SWLB medium with or without the L-tyrosine or D-tyrosine at a concentration of 1 mg/mL. Total RNAs was isolated using Tiangen RNA prepPure Cell (Tigangen Biotech, Beijing, China) following the manufacturer’s instructions. Complementary DNA was synthesized using the Reverse Transcription System (Promega, Madison, WI, United States) according to manufacturer’s instructions. qRT-PCR was performed using the Step One Real-Time PCR system (Life Technologies, Carlsbad, CA, United States) using SYBR Green Mix (Thermo Fisher Scientific, Waltham, MA, United States). Primers for qRT-PCR for *rrsG*, *melA*, *hsp90, hmgA*, *fahA*, and *maiA* are listed in Supplementary Table S1. The transcript of the *rrsG* was used to normalize the total RNAs in different samples. A quantification method based on the relative amount of a target gene *versus* a reference gene was used (Pfaffl, 2001). Fold change of the target gene at different temperatures (T1 and T2) was calculated as 2ˆ-(C_t_ *_target__*_T1_-C_t_ *_rrsG_*__T1_)/2ˆ-(C_t_ *_target_*__T2_-C_t_ *_rrsG_*__T1_). The level of *rrsG* transcript was used to normalize the gene expression data in different samples.

### Heat Stress Assay

For wild-type SM9913 and *melA* mutant strains, cells were grown in SWLB medium and treated at 45°C for 30 min in the exponential stage (turbidity ∼1.0 at OD_600_). Cell viability was determined by serial dilutions using 3.4% NaCl solution and plated on SWLB agar plates (Donegan et al., 1991). For *E. coli* K12 with an empty vector or expressing the *melA* product from SM9913, cells were grown in LB medium supplemented with 30 μg/mL chloramphenicol and 0.5 mM IPTG and treated at 65°C for 10 min in the exponential stage (turbidity ∼1.0 at OD_600_). Cell viability was determined by serial dilutions using 0.8% NaCl solution and plated on LB agar plates.

## Results

### SM9913 Forms Biofilm

**FIGURE 1:**
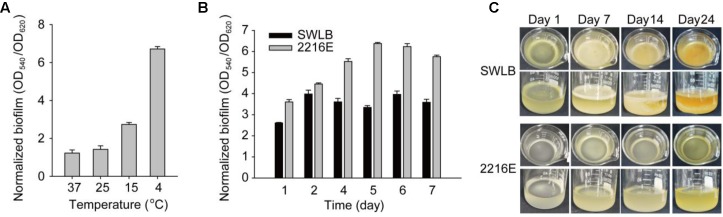
SM9913 forms attached biofilm and pellicle. **(A)** Attached biofilm formation of SM9913 cultured at different temperatures for 24 h in SWLB medium. **(B)** Attached biofilm formation of SM9913 cultured in SWLB and 2216E at 15°C. Data represent the average of 15 replicate wells from three independent cultures, and one standard deviation is shown in both **(A,B)**. **(C)** Pellicle formation of SM9913 cultured without shaking in SWLB and 2216E at 15°C. Three independent cultures were tested and one representative image is shown.

Attached biofilm formation was examined using the 96-well polystyrene plate assay, which partially mimics an aquatic environment in which bacteria are attached to the surface of the particles (Lee et al., 2008). For clarity, we hereafter refer to this type of biofilm as the “attached biofilm.” SM9913 grew at a wide range of temperatures from 4 to 37°C (see growth curve in SWLB at 15, 25, and 37°C in Supplementary Figure S1). Since the optimal growth temperature of SM9913 is 15°C (Qin et al., 2011), and the doubling time of SM9913 was over 30 h in SWLB at 4°C, 15°C was chosen as the optimal temperature in this study. Attached biofilm of SM9913 was analyzed in a nutrient-enriched medium (sea water Luria-Bertani; SWLB), in which it formed attached biofilm after 1 day of incubation at 4, 15, 25, and 37°C. Results showed that SM9913 formed the most abundant attached biofilm when cultured at 4°C (**Figure 1A**). In SWLB or a nutrient-less marine medium 2216E, SM9913 formed an initial attached biofilm within 1 day, which continued to grow until day 5 at the optimal growth temperature (**Figure 1B**). Moreover, SM9913 formed 1.9 ± 0.1-fold more attached biofilm in 2216E compared to SWLB after 5 days of incubation (**Figure 1B**), suggesting lower nutrient levels led to the formation of more attached biofilm. Static culturing of SM9913 in aerobic conditions can also lead to the formation of “pellicle” on the surface of static liquid medium, resembling the biofilm that formed at the liquid–air interface; for clarity, this type of biofilm is henceforth referred to as “pellicle.” Visible pellicle was observed after 1 day incubation of SM9913 at 15°C in SWLB, continued to grow until day 6 before gradually dispersed over time (**Figure 1C**). In contrast to attached biofilm formed at the solid–liquid interface, SM9913 formed pellicle in SWLB but not in 2216E over a period of 2 weeks, indicating that higher nutrient levels promoted pellicle formation (**Figure 1C**). Therefore, SM9913 has the capacity to form biofilm at both the solid–liquid and air–liquid interfaces, with lower temperatures and lower nutrient levels promoting the formation of attached biofilm and higher nutrient levels promoting the formation of pellicles at the air–liquid interface.

### Pyomelanin Production Is Induced at Higher Temperatures

**FIGURE 2:**
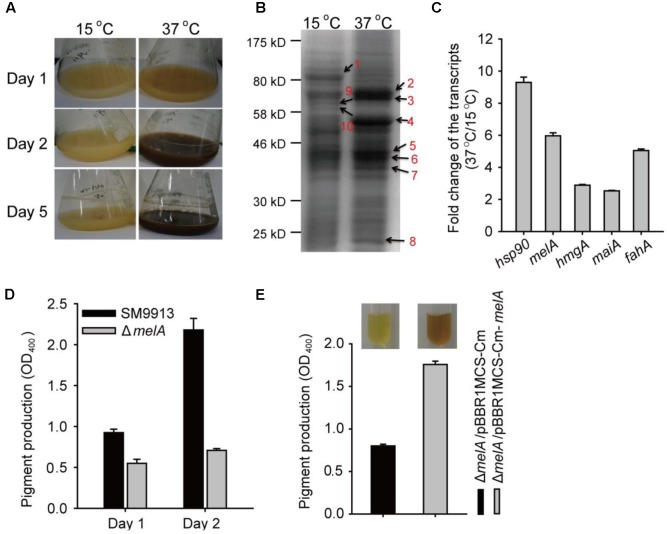
Pyomelanin production is induced at higher temperatures in SM9913. **(A)** Pigment production of SM9913 cultured in SWLB at different temperatures on days 1, 2, and 5. Three independent cultures were tested and one representative image is shown. **(B)** Protein profiles of SM9913 grown at 15 and 37°C for 24 h in SWLB. Arrows indicate the protein band that selected for mass spectrometry analysis. **(C)** Fold change of the *hsp90*, *melA*, *hmgA*, *maiA*, and *fahA* transcription in SM9913 at 37°C compared to the condition at 15°C quantified by qRT-PCR. Overnight cultures were diluted to OD_600_∼0.1 and re-grown until OD_600_∼1 at 15°C. Cells were collected before and after transfer to 37°C for 25 min. **(D)** Pigment production of SM9913 and Δ*melA* strains cultured at 37°C. **(E)** Pigment production of SM9913 and Δ*melA* strains with exogenous SM9913 *melA* gene expression (Δ*melA*/pBBR1MCS-Cm-*melA*) or with an empty plasmid (Δ*melA*/pBBR1MCS-Cm) at 37°C for 2 days measured at OD_400_. Data are the average of three independent cultures, and one standard deviation is shown in **(C–E)**.

Wild-type SM9913 strain produced a dark brown pigment when grown in SWLB or 2216E at higher temperatures after prolonged incubation (e.g., 37°C for 2 days) (**Figure 2A**, only the culture grown in SWLB was shown here). To explore the molecular basis of the occurrence of the dark brown pigment at higher temperatures, cells grown at 37 and 15°C were collected and the protein profiles were assessed by SDS-PAGE. A total of 10 differentially produced bands of various sizes were selected for mass spectrometry analysis (**Figure 2B** and Supplementary Table S2). Among them, two of the proteins induced at 37°C were identified to be 4-hydroxyphenylpyruvate dioxygenase (4-HPPD) (band #7, ∼38 kDa) (Supplementary Figure S2) and heat shock protein 90 (HSP90) (band #3, ∼70 kDa). HSP90 is a chaperone protein that help other protein folding properly, and stabilizes proteins against heat damage (Rutherford and Lindquist, 1998). 4-HPPD is encoded by *melA* gene which catalyzes the conversion of 4-hydroxyphenylpyruvate into HGA during the catabolism of L-tyrosine. HGA can be auto-oxidized, and polymerized to form pyomelanin (Moran, 2005). The 4-HPPD protein of SM9913 (PSM_A0972) shares 72% similarity (100% coverage) with the 4-HPPD protein of *Shewanella oneidensis* (Supplementary Figure S3), which is a key protein participate pyomelanin biosynthesis in *S. oneidensis* (Turick et al., 2009). To further verify that the pigment induced at 37°C was pyomelanin, we performed FTIR to analyze the structure of the pigment which has been described previously for pyomelanin (Bilinska, 1996). Results showed that the mainly FTIR bands at the waves of 3, 282, 2, 926, 1, 519 cm^-1^ as well as the fingerprint regions between 1, 000 and 500 cm^-1^ shared highly similarity between the pigment extracts of SM9913 to the FTIR scans of pyomelanin as previously reported from *Shewanella algae* (Turick et al., 2002) and *Aspergillus fumigatus* (Schmaler-Ripcke et al., 2009) (Supplementary Figure S4). In addition, synthetic pyomelanin produced by using commercially purchased HGA (Tokyo Chemical Industry, Co., Ltd, Japan) to synthesize pyomelanin via *in vitro* auto-oxidation was analyzed by FTIR and they also shared high similarity with the pigment extracted from SM9913 (Supplementary Figure S5).

Consistent with increased protein expression of 4-HPPD and HSP90 at 37°C, the transcription of *melA* and *hsp90* were induced upon the temperature upshift by sixfold and ninefold, respectively (**Figure 2C**). In order to investigate the physiologic role of *melA* in SM9913, the *melA* coding region was removed from the SM9913 genome using the fusion PCR method which we developed for genetic manipulation of *Pseudoalteromonas* strains (Wang et al., 2015). Deletion of *melA* gene was verified by PCR followed by DNA sequencing (Supplementary Figure S6A). As expected, no visible dark brown pigment was produced in the Δ*melA* strain after prolonged incubation at 37°C for up to 3 days (Supplementary Figure S6B). The level of pyomelanin in the culture was also quantified by measuring the absorbance of the supernatant at 400 nm, a method employed previously to measure the pyomelanin production in bacterial culture (Turick et al., 2002). As shown in **Figure 2D**, the Δ*melA* strain produced a reduced level of pyomelanin in the culture than that of the wild-type strain when cultured at 37°C, confirming that *melA* expression was critical for pyomelanin production at higher temperatures. In addition, we performed the complementation study by constructing the pBBR1MCS-Cm-*melA* plasmid to express the *melA* gene using its native promoter. As expected, the introduction of pBBR1MCS-Cm-*melA* into the Δ*melA* strain restored the pigmentation at 37°C, while the introduction of the empty plasmid pBBR1MCS-Cm could not do so (**Figure 2E**). Collectively, these results demonstrate that the accumulation of the *melA* product increased the pyomelanin production in SM9913 at higher temperatures.

### Pyomelanin Production Increases during Pellicle Formation

**FIGURE 3:**
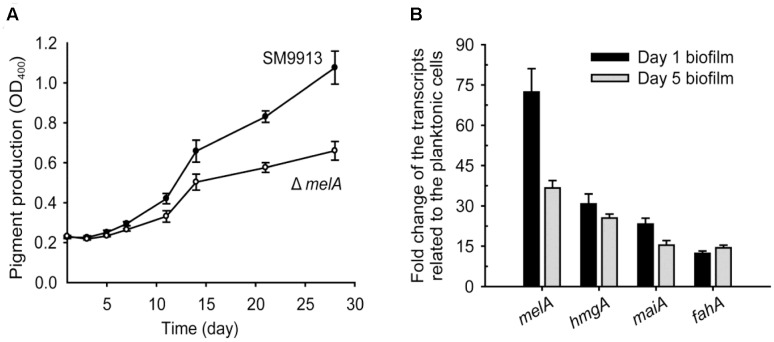
Pyomelanin production is increased during biofilm formation. **(A)** Pigment production of SM9913 and Δ*melA* strains were measured by OD_400_ in static culture at 15°C in SWLB. **(B)** Fold changes of the *melA*, *hmgA*, *maiA*, and *fahA* transcription in biofilm cells (day 1 or day 5) compared to planktonic cells (OD_600_∼ 1) in SWLB at 15°C. Data are the average of three independent cultures, and one standard deviation is shown in **(A,B)**.

During the formation of pellicles in SM9913, an increased amount of brownish pigment at later-stage was observed (**Figure 1C**). We therefore hypothesized that pyomelanin production is increased during the formation of pellicles in SM9913. In order to test this hypothesis, supernatants were collected during the formation of pellicle and quantified by measuring the absorbance at 400 nm. The OD_400_ values of the wild-type strain was increased during the culturing, while the Δ*melA* strain produced a reduced level of pigment (**Figure 3A**), suggesting an increased production of pyomelanin in the culture of SM9913. We further performed qRT-PCR to analyze the *melA* transcription during pellicle formation. Notably, when cultured at the optimal temperature of 15°C, the transcription of *melA* was increased 72 ± 8-fold at the initial stage of pellicle formation (day 1), and 36 ± 3-fold at the later stage of pellicle formation (day 5) compared to the cells growing planktonically (**Figure 3B**). Similar results were obtained during pellicle formation at 4°C, with the transcription of *melA* significantly induced at day 5 at the onset of forming visible pellicle (Supplementary Figure S7). Therefore, pyomelanin production was increased during pellicle formation of SM9913.

### Pyomelanin Formation Increases Heat Resistance

**FIGURE 4:**
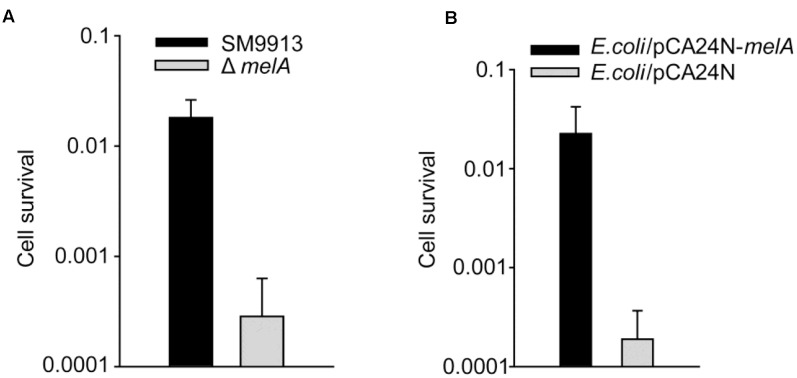
Pyomelanin production requires *melA* and the *melA* product increases temperature tolerance. **(A)** The cell viability of SM9913 and Δ*melA* strains after heat stress of 45°C for 30 min. **(B)** The cell viability of *Escherichia coli* K12/pCA24N-*melA* and *E. coli* K12/pCA24N after heat stress of 45°C for 30 min. Expression of *melA* in *E. coli* K12 was induced with 0.5 mM IPTG. Data are the average of three independent cultures, and one standard deviation is shown in **(A,B)**.

We next tested whether the formation of pyomelanin protects SM9913 from stress. When treated at 45°C for 30 min, the cell viability of Δ*melA* strain decreased 63 ± 1-fold compared to wild-type strain (**Figure 4A**). To examine whether ectopic expression of the *melA* product can provide protection for other bacterial cells during heat stress, the *melA* gene from SM9913 genome was cloned and expressed in *E. coli* K12 strain (**Table 1**). *E. coli* K12 carries a tyrosine aminotransferase which is required for the initial conversion of tyrosine to 4-hydroxyphenylpyruvate (Gelfand and Rudo, 1977), but it lacks the gene/genes responsible for conversion of 4-hydroxyphenylpyruvate to HGA which is required for the synthesis of pyomelanin. The ectopic expression of the *melA* product in *E. coli* K12 was checked by SDS-PAGE and a protein band with a similar size of 4-HPPD was produced as expected (Supplementary Figure S8A). When *melA* was induced by adding IPTG in *E. coli* K12, a dark brown pigment was observed in the culture supernatant (Supplementary Figure S8B). Moreover, when treated at 65°C for 10 min, the cell viability of *E. coli* K-12 increased 119 ± 1-fold when *melA* was induced (**Figure 4B**). Thus, the synthesis of pyomelanin also plays a protective role against heat damage in *E. coli*.

### Genes that Involved in L-Tyrosine Catabolism Are Differentially Regulated

**FIGURE 5:**
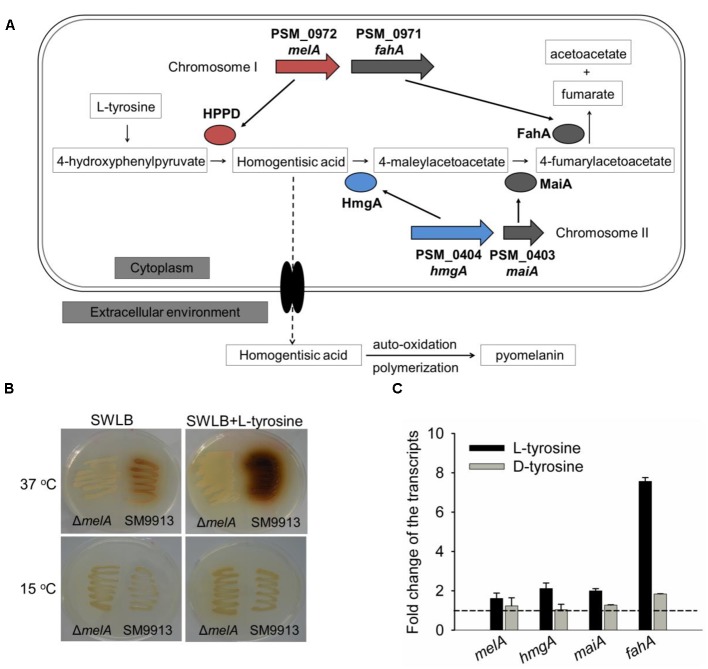
Key enzymes involved in L-tyrosine catabolism in SM9913. **(A)** Predicted catabolic pathway of L-tyrosine and representative loci in the SM9913 genome. The predicted enzymes HPPD, HmgA, FahA, and MaiA encoded by *melA*, *hmgA*, *fahA*, and *maiA* located on the two different chromosomes are shown. The accumulation of HGA could secret to extracellular environment and the auto-oxidization of HGA lead to the production of pyomelanin. **(B)** Pigmentation of SM9913 and Δ*melA* strains grown on SWLB agar plates or SWLB agar plates supplemented with 1 mg/mL L-tyrosine at 15 or 37°C, respectively. Three independent cultures were tested and one representative image is shown. **(C)** Fold changes of the *melA*, *hmgA*, *maiA*, and *fahA* transcription with or without L-tyrosine or D-tyrosine supplementation. Data are the average of three independent cultures, and one standard deviation is shown.

Pyomelanin was produced by the accumulation of HGA starting from the L-tyrosine degradation (Kotob et al., 1995; Serre et al., 1999). Genomic analysis suggests that all of the four genes that participate in the L-tyrosine catabolism (as defined in the KEGG database^1^) are present in the genome of SM9913. For many bacteria with one chromosome, the genes that participate in the L-tyrosine catabolism were located in the chromosome as one single transcriptional unit, such as in *Pseudomonas aeruginosa* PAO1 and *P. aeruginosa* PA14 (Rodriguez-Rojas et al., 2009). However, in SM9913, the genes involved in the catabolism of L-tyrosine are located on two chromosomes, respectively. Gene *fahA*, located 197 bp upstream of *melA* on chromosome I, encodes fumarylacetoacetase which converts 4-fumarylacetoacetate into acetoacetate and fumarate. Two other genes, *hmgA* (PSM_B0404) and *maiA* (PSM_B0403), are located on chromosome II, with *maiA* lying 7 bp upstream of *hmgA*. Gene *hmgA* encodes for homogentisate 1, 2-dioxygenase, which converts HGA to 4-maleylacetoacetate. Gene *maiA* encodes maleylacetoacetate isomerase, which converts 4-maleylacetoacetate to 4-fumarylacetoacetate (Flydal et al., 2012) (**Figure 5A**). To test whether L-tyrosine was required to the formation of pyomelanin, L-tyrosine was added to the SWLB agar plates and pyomelanin production was investigated. As expected, the addition of L-tyrosine increased the pyomelanin production in the wild-type strain at 37°C, while did not induce the formation of pyomelanin after prolonged incubation at 15°C (**Figure 5B**). qRT-PCR results showed that the addition of L-tyrosine increased the transcripts of all of the four genes, and the transcription of *melA* was slightly lower than that of *hmgA* and *maiA* (**Figure 5C**). Importantly, *fahA* was the most highly induced gene among these four genes at 15°C (**Figure 5C**). High expression of *fahA* product can convert more 4-fumarylacetoacetate into acetoacetate and fumarate. Acetoacetate and fumarate could therefore be utilized in the TCA cycle and contribute to the generation of ATP. However, under heat stress and during biofilm formation condition in which increased pyomelanin production was detected, the four genes were all induced and the level of *melA* transcription which is responsible for the synthesis of HGA was significantly higher induced than those of the *hmgA* and *maiA* which are responsible for the degradation of HGA (**Figures 2C, 3B**). Collectively, these results demonstrated that the formation of pyomelanin in SM9913 is mainly caused by an increased expression of *melA* gene which is responsible for the synthesis of HGA as comparing to the genes that are responsible for the degradation of HGA during heat stress and during biofilm formation.

## Discussion

In this study, we found that SM9913 has the ability to form attached biofilm on the inorganic surface and to form pellicles growing at the air–liquid interface. Biofilm formation may benefit bacteria and other biofilm-forming organisms to survive in different marine habitats, including the deep-sea sediment (Hall-Stoodley et al., 2004). Some *Pseudoalteromonas* species are known to form biofilms (Mai-Prochnow et al., 2004; Huang et al., 2007; Iijima et al., 2009), and the pigmented *Pseudoalteromonas* spp. has been shown to exhibit antifouling and antibacterial capabilities (Egan et al., 2002; Bowman, 2007). In this study, we showed that the deep-sea non-pigmented bacteria *Pseudoalteromonas* sp. SM9913 produced pyomelanin pigment only at elevated temperatures or during biofilm formation. We also have demonstrated here that *melA* gene which is responsible for the synthesis of HGA was induced by a high temperature and during biofilm formation. The pyomelanin production was abolished when the *melA* gene was deleted. Additionally, deletion of *melA* in SM9913 reduced survival upon heat stress. Ectopic expression of the *melA* gene of SM9913 in *E. coli* K-12 which does not have an endogenous *melA* gene was also able to provide protection to *E. coli* cells during heat stress.

The physiological function of pyomelanin has been previously explored in the clinically important pathogens. For example, in *Legionella pneumophila*, the secreted pyomelanin conferred a ferric reductase activity which played an important role in iron uptake, thus enhancing the growth under iron-limiting conditions (Chatfield and Cianciotto, 2007; Zheng et al., 2013). In *Pseudomonas aeruginosa* and *Vibrio cholerae*, pyomelanin producing or hyperproducing variants were frequently isolated and showed increased persistence or virulence (Rodriguez-Rojas et al., 2009; Valeru et al., 2009). In *Burkholderia cenocepacia*, pyomelanin production helped scavenge free radicals, resulting in the attenuation of the host cell (Keith et al., 2007). Although SM9913 was isolated from the deep-sea sediment where the temperature was near the freezing-point, it retained the ability to survive at a wide range of temperatures. In this study, we found that the pyomelanin was induced during heat stress and it is possible that the process of auto-oxidizing HGA to pyomelanin might help to scavenge free radicals, which are often generated during heat stress, and to protect the cells from heat damage (Benov and Fridovich, 1995; Mols and Abee, 2011). The increased expression of *melA* gene is responsible for the synthesis of pyomelanin at elevated temperatures or during biofilm formation; however, we observed that the *melA* mutant strain still showed a small increase by the absorbance at 400 nm during heat stress and during biofilm formation (**Figures 2D, 3A**). It is possible that the other type of pigments may be produced under heat stress and during pellicle formation through an unknown pathway, which showed absorbance at 400 nm in the culture supernatants. Whether the production of pyomelanin or other unknown pigments can be induced in other stress conditions needs further investigation. Meanwhile, the physiological function of pyomelanin in extreme marine environment also needs to be explored.

Although it has been suggested that the *Pseudoalteromonas* spp. can be categorized into two clades, we tend to believe that this classification needs refinement, especially for pyomelanin-producing strains. Most of the pigments, such as prodiginines (red) from *Pseudoalteromonas rubra* (Feher et al., 2008), cyclodigiosin hydrochloride (red) from *Pseudoalteromonas denitrificans* (Kim et al., 1999), violacein (purple) from *Pseudoalteromonas luteoviolacea* (Yang et al., 2007), and tambjamines-like alkaloid (yellow) from *Pseudoalteromonas tunicata* (Franks et al., 2005), are synthesized by specific enzymes that are only present in the genome of their respective strains. However, the L-tyrosine catabolic genes cluster, including *melA*, *hmgA*, *fahA* and *maiA* genes, that is responsible for the production of pyomelanin are found in all sequenced *Pseudoalteromonas* strains (data not shown). Thus, we reasoned that *Pseudoalteromonas* spp. are capable of producing pyomelanin when encounter certain stress condition, which help the bacteria to adapt to changing marine environment. Three *Pseudoalteromonas* species have been characterized as melanin-like pigments-producing strains, including *Pseudoalteromonas nigrifaciens* (Baumann et al., 1984), *Pseudoalteromonas aliena* (Ivanova et al., 2004), and *Pseudoalteromonas distinct* (Ivanova et al., 2004), but it remains to be determined whether the production of dark brown pigments is due to the accumulation of 4-HPPD or the defective HmgA activity who is responsible for the degradation of HGA. Indeed, strains that overproducing pyomelanin were commonly isolated from patients with cystic fibrosis and bronchiectasis, in which *Pseudomonas aeruginosa* living in biofilms, and the hyper-production of pyomelanin in these mutants were mostly due to the loss activity of HmgA (Rodriguez-Rojas et al., 2009; Hocquet et al., 2016). Naturally-occurring hyper-pyomelanin producing mutants of *Vibrio cholerae* due to the mutation of *hmgA* also have been isolated from different environmental waters or patients (Wang et al., 2011). Moreover, we have recently isolated a pyomelanin-producing variant from the biofilm cells of a non-pigmented strain *Pseudoalteromonas lipolytica* that are caused by the point mutation in *hmgA* gene (data not shown here). Thus, a detailed genetic analysis of L-tyrosine catabolic pathway genes in *Pseudoalteromonas* strains is needed to elucidate the exact mechanism underlying the production of melanin-like compound. Further effort is needed to explore the exact mechanism controlling the L-tyrosine catabolism under different stress conditions in different organisms.

## Author Contributions

XW conceived the idea and designed the project. ZZ, XC, PW, YG, XL, and BL carried out the experiments. XW, ZZ, and XC analyzed data. XW, ZZ, and XC wrote the manuscript with input from all other authors.

## Conflict of Interest Statement

The authors declare that the research was conducted in the absence of any commercial or financial relationships that could be construed as a potential conflict of interest.

## Footnotes

1 http://www.kegg.jp/
