# Supplementary material for: Biofilm Formation and Heat Stress Induce Pyomelanin Production in Deep-Sea Pseudoalteromonas sp. SM9913
Source: Front Microbiol. 2017 Sep 21;8:1822. doi: 10.3389/fmicb.2017.01822 (PMC5613676; doi:10.3389/fmicb.2017.01822)
Supplement: Supplementary file 1 [file Data_Sheet_1.DOCX]

**Supplementary File**

**Biofilm Formation and Heat Stress Induce Pyomelanin Production in Deep-sea *Pseudoalteromonas* sp. SM9913**

**Zhenshun Zeng^1†^, Xingsheng Cai^1†^, Pengxia Wang^1^, Yunxue Guo^1^, Xiaoxiao Liu^1^, Baiyuan Li^1,2^, Xiaoxue Wang^1*^**

^1^Key Laboratory of Tropical Marine Bio-resources and Ecology, the South China Sea Institute of Oceanology, Chinese Academy of Sciences, Guangzhou, 510301, P. R. China
^2^Guangdong Provincial Key Laboratory of Microbial Culture Collection and Application, Guangdong Institute of Microbiology, Guangzhou, 510301, P. R. China

^†^These authors contribute equally to this work.

***Correspondence:** Xiaoxue Wang, E-mail: [xxwang@scsio.ac.cn](mailto:xxwang@scsio.ac.cn); Tel:+ 86 20 89267515; Fax: +86 20 89235490

**Table S1.** Nucleotide sequences of the primers or primer sets used in this study. F indicates forward primer and R indicates reverse primer. The restriction enzyme sites are boxed, and the corresponding restriction enzymes were shown in brackets.

| **Primer Name/Purpose** | **Primer sequence (listed 5’ to 3’)** | |
| --- | --- | --- |
| **qRT-PCR** | |  |
| *melA* | | GCGCGTAGAGAACGCTCAAG |
|  |  | ATTAACGACGTTTTGCTCAGCTAA |
| *hmgA* | | GTTGTTGCTTGGACAGGCAAT |
|  |  | GGGCGGAAGGTGTTTTCAG |
| *fahA* | | GACTGTGGCACAAATGGTGACT |
|  |  | TCACGTTGTCGCCGTCTTC |
| *maiA* | | CAATACCCTGAAACACCATTGCT |
|  |  | AACCCAGTGGCGGTACCAT |
| *hsp*90 | | CATTAACCGTGCTACGGCATT |
|  |  | GCTCGCGGTTCCATAAATCA |
| *rrsG* | | TGATAAACCGGAGGAAGGTG |
|  |  | TTCATGGAGTCGAGTTGCAG |
| **Construction of in-frame deletion mutants** | | |
| melA-up-F | | ACATGCATGCTCTTACACACAGTGGGTGAGC |
| melA-up-R | | CGGAATTCTATAATTACTCCCGCAATTAAGC |
| melA-down-F | | CGGAATTCTTAAAATTATGACTCTAAAAAGCAGCG |
| melA-down-R | | AGCGTCGACCAGCTCAAGCTCGTAATCAAGAC |
| *melA*-F | | GCACTTCCATCTCGCTACCT |
| *melA*-R | | AATAAGAACATAGCACCAGCAAG |
| pK18-F | | ATTCCGCTGGCAGCTTAAG |
| pK18-R | | GGTAACGCCAGGGTTTTCC |
| **Construction of expression vectors** | | |
| pCA24N-*melA*-F | | GCCAGTGAAGTAAATAACCCTTT |
| pCA24N-*melA*-R | | GGTAAAACGCCGCGGCGTTCTTG |
| pCA24N-F | | GCCCTTTCGTCTTCACC |
| pCA24N-R | | CCGGCATCCGCTTACAGAC |
| SM9913-*melA*-F | | CCGGAATTCTGGTCTCGCCTACTTACAGCGT |
| SM9913-*melA*-R | | CCGCTCGAGTTATAAAACGCCGCGGCG |
| pBBR1MCS-F | | TCGTTAAATAGCCGCTTATG |
| pBBR1MCS-R | | AATTTCACACAGGAAACAGC |

**Table S2.** Sequencing of protein bands identified to be differentially produced at 37 ^o^C *versus* at 15 ^o^C in SM9913 by mass spectrometry. Hits are indicated the numbers of the digested peptides that match the target amino acid sequence of the related protein.

| **Band** | **Products** | **Locus tag** | **Length (aa)** | **Hits** |
| --- | --- | --- | --- | --- |
| **# 1** | TonB-dependent receptor | PSM_A2033 | 925 | 40 |
|  | TonB-dependent receptor | PSM_A1222 | 895 | 10 |
|  | hypothetical protein PSM_A2680 | PSM_A2680 | 915 | 6 |
|  | T6SS ATPase | PSM_B0219 | 872 | 4 |
| **# 2** | TonB-dependent siderophore receptor | PSM_A3122 | 709 | 9 |
|  | TonB-dependent receptor | PSM_A0116 | 691 | 8 |
|  | methyl-accepting chemotaxis protein | PSM_B0197 | 705 | 7 |
|  | ligand-gated channel protein | PSM_A0536 | 718 | 5 |
| **# 3** | **heat shock protein 90** | **PSM_A1820** | **637** | **9** |
|  | peptidase S9 prolyl oligopeptidase | PSM_B0288 | 682 | 7 |
|  | UvrD family helicase | PSM_A0670 | 638 | 6 |
|  | acetyl-coenzyme A synthetase | PSM_A2364 | 646 | 4 |
| **# 4** | dihydrolipoamide acetyltransferase | PSM_A1603 | 522 | 3 |
|  | hypothetical protein | PSM_A3028 | 592 | 2 |
|  | urocanate hydratase | PSM_A0287 | 559 | 2 |
|  | preprotein translocase subunit YidC | PSM_A3124 | 544 | 2 |
| **# 5** | phosphoribosylamine--glycine ligase | PSM_A2747 | 428 | 5 |
|  | Xaa-Pro dipeptidase | PSM_A0567 | 433 | 2 |
|  | aminopeptidase PepB | PSM_A2450 | 432 | 2 |
|  | fumarylacetoacetase | PSM_A0971 | 437 | 2 |
| **# 6** | putative AcnD-accessory protein | PSM_A1294 | 391 | 5 |
|  | hypothetical protein | PSM_A1833 | 419 | 4 |
|  | 3-oxoacyl-ACP synthase | PSM_A2145 | 403 | 2 |
| **# 7** | **4-hydroxyphenylpyruvate dioxygenase** | **PSM_A0972** | **348** | **2** |
|  | glycine C-acetyltransferase | PSM_A0770 | 398 | 2 |
|  | acyl-CoA dehydrogenase | PSM_A1501 | 385 | 1 |
| **# 8** | hypothetical protein | PSM_A1581 | 270 | 10 |
|  | 6-phosphogluconolactonase | PSM_A1886 | 236 | 5 |
|  | enoyl-CoA hydratase | PSM_A1502 | 259 | 5 |
|  | hypothetical protein | PSM_A2148 | 211 | 3 |
|  | enoyl-CoA hydratase/isomerase | PSM_A1495 | 256 | 3 |
|  | ThiJ/PfpI protein | PSM_A0948 | 227 | 2 |
| **# 9** | hypothetical protein | PSM_A2278 | 671 | 20 |
|  | peptidase S9 prolyl oligopeptidase | PSM_B0288 | 682 | 12 |
|  | peptidase M13 | PSM_A0280 | 690 | 10 |
|  | UvrD family helicase | PSM_A0670 | 638 | 8 |
| **# 10** | beta-ketoacyl synthase | PSM_A1416 | 635 | 16 |
|  | N-6 DNA methylase | PSM_A2853 | 559 | 9 |
|  | dihydrolipoamide acetyltransferase | PSM_A1603 | 522 | 8 |

**
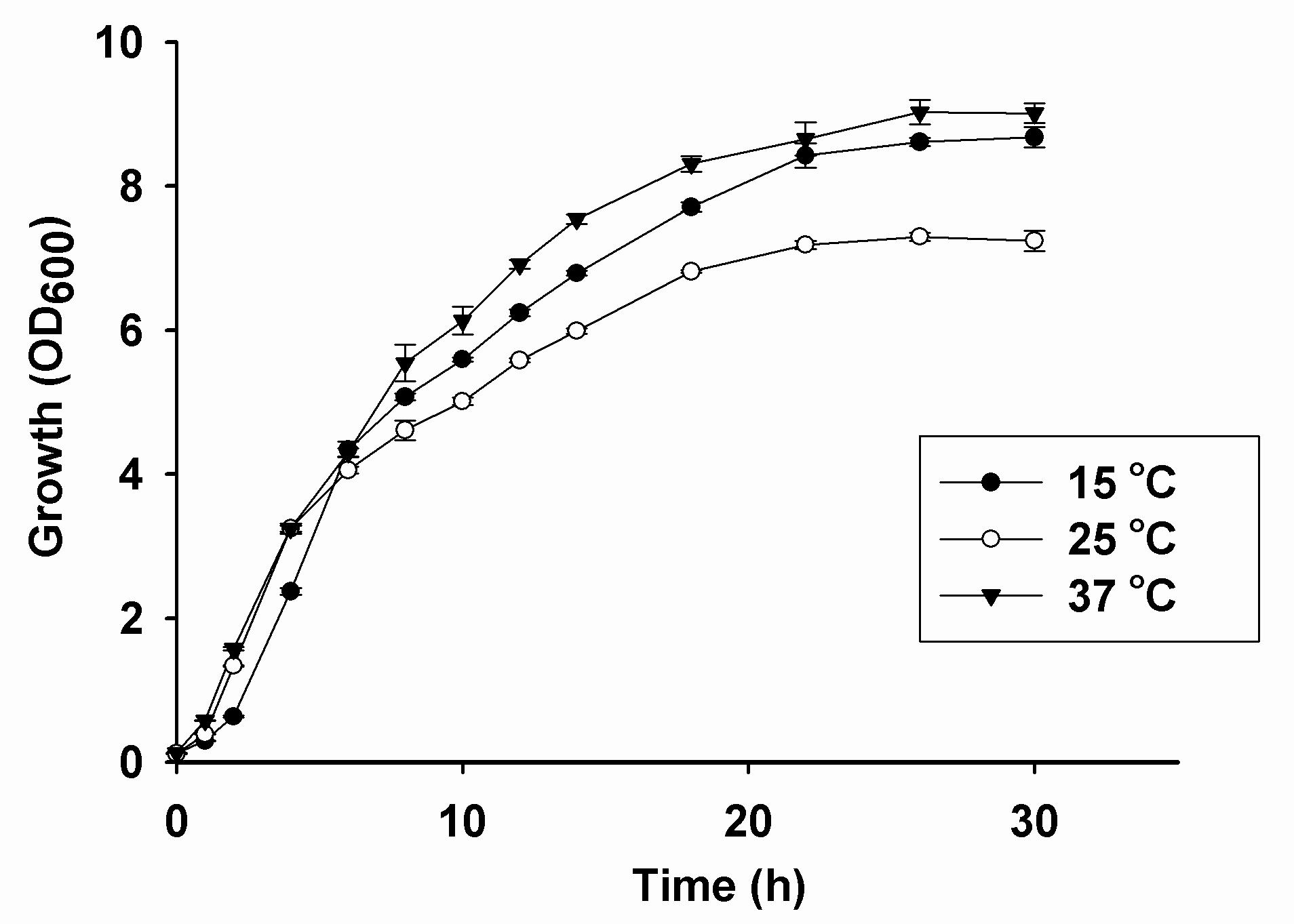
**

**Figure S1.** Growth curve of SM9913 in SWLB at different temperature.


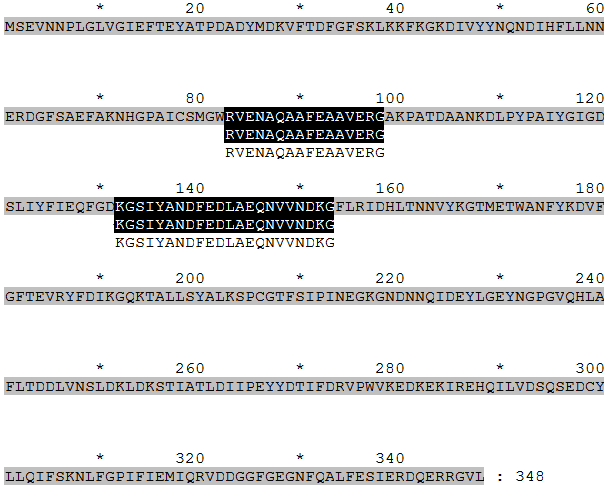


**Figure S2.** SM9913 HPPD peptide sequences identified by ms/ms analysis. Two digested peptides (highlight in black) are matched with the HPPD, encoded by *melA*, in SM9913


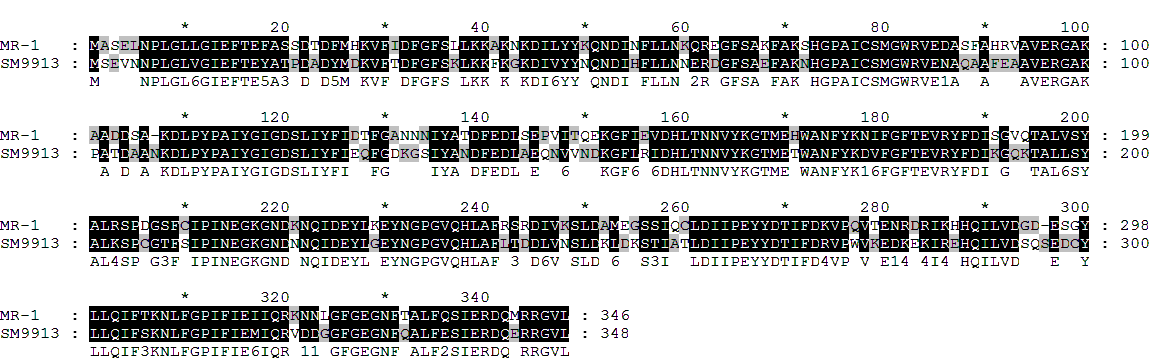


**Figure S3.** *melA* from SM9913 shows 72% identity with *Shewanella oneidensis* MR-1 *melA* at the amino acid level.

**Figure S4.** FTIR spectrum of the pigment extracted from SM9913 cultures induced by high temperature.

**Figure S5.** FTIR spectrum of the synthetic pyomelanin from auto-oxidation of the homogentisic acid.

**
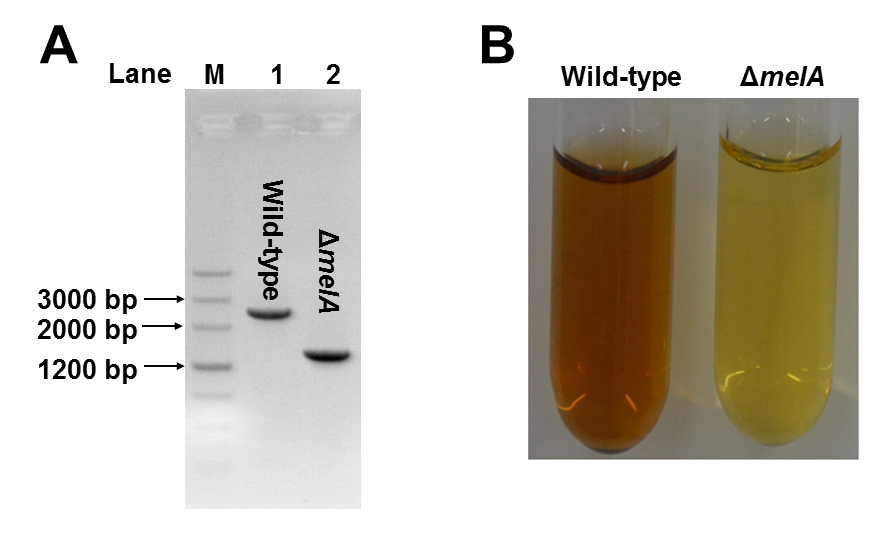
**

**Figure S6. (A)** PCR confirmation of the removal of the *melA* gene from the wild-type SM9913 genome using the melA-F and melA-R primer pair. **(B)** Supernatants extracted from the wild-type SM9913 and Δ*melA* strain when incubated at 37 °C for 3 days.


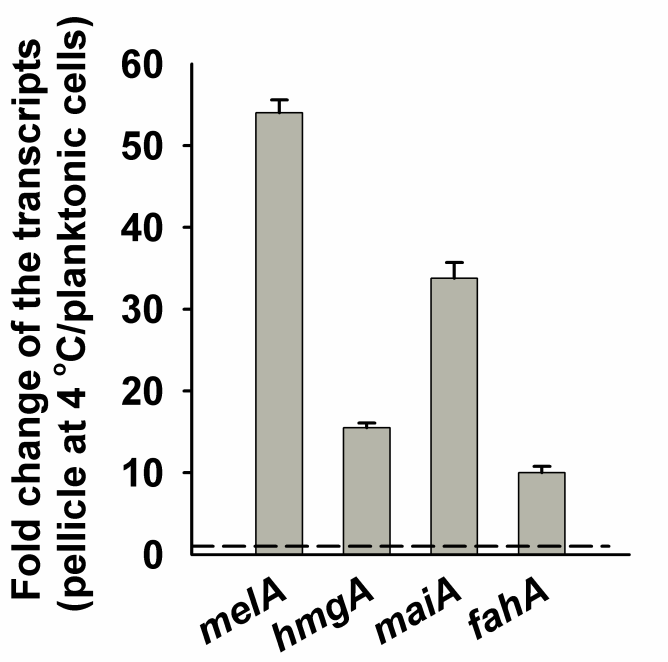


**Figure S7.** Fold changes of *melA*, *hmgA*, *maiA*, and *fahA* transcription in day 5 biofilm cells at 4 °C compared to planktonic SM9913 cells (OD_600_~ 1) at 15 °C in SWLB. Data are the average of three independent cultures, and one standard deviation is shown.


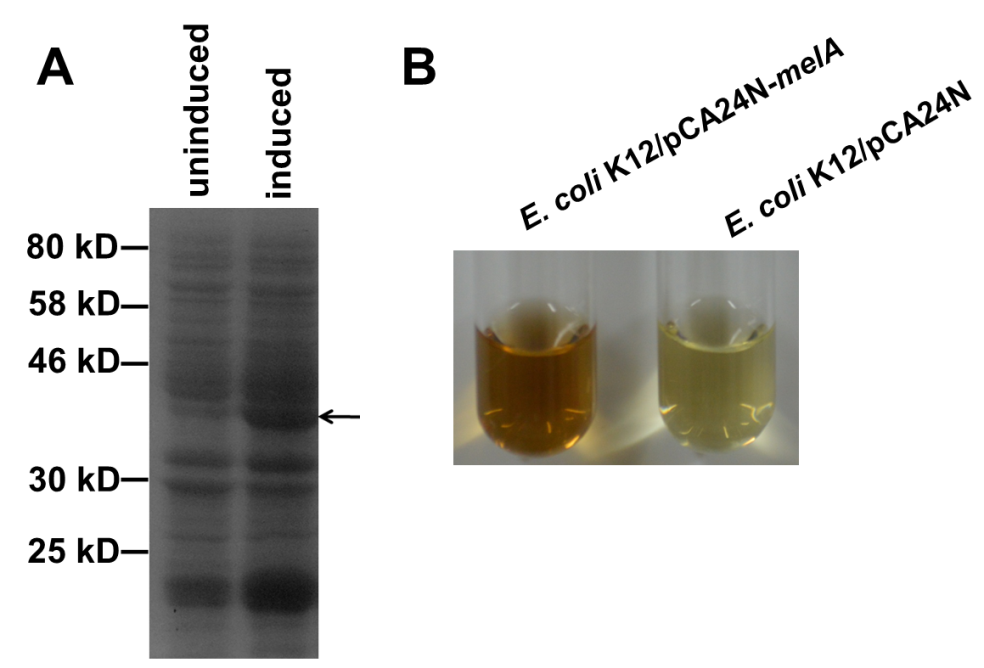


**Figure S8. (A)** SDS-PAGE analysis of *E. coli* K12/pCA24N-*melA* with IPTG (induced) and without IPTG (uninduced). Predicted HPPD band was indicated with an arrow. (**B**) Color morphology of *E. coli* K12/pCA24N-*melA* and *E. coli* K12/pCA24N strains with IPTG, respectively.
